# The clonal relation of primary upper urinary tract urothelial carcinoma and paired urothelial carcinoma of the bladder

**DOI:** 10.1002/ijc.33327

**Published:** 2020-10-13

**Authors:** Thomas van Doeveren, Jose A. Nakauma‐Gonzalez, Andrew S. Mason, Geert J. L. H. van Leenders, Tahlita C. M. Zuiverloon, Ellen C. Zwarthoff, Isabelle C. Meijssen, Angelique C. van der Made, Antoine G. van der Heijden, Kees Hendricksen, Bas W. G. van Rhijn, Charlotte S. Voskuilen, Job van Riet, Winand N. M. Dinjens, Hendrikus J. Dubbink, Harmen J. G. van de Werken, Joost L. Boormans

**Affiliations:** ^1^ Department of Urology, Erasmus MC Cancer Institute University Medical Center Rotterdam The Netherlands; ^2^ Department of Medical Oncology, Erasmus MC Cancer Institute University Medical Center Rotterdam The Netherlands; ^3^ Cancer Computational Biology Center, Erasmus MC Cancer Institute University Medical Center Rotterdam The Netherlands; ^4^ Jack Birch Unit for Molecular Carcinogenesis, Department of Biology The University of York York UK; ^5^ York Biomedical Research Institute The University of York York UK; ^6^ Department of Pathology, Erasmus MC Cancer Institute University Medical Center Rotterdam The Netherlands; ^7^ Department of Urology, Radboud University Medical Center Radboud Institute for Health Sciences Nijmegen The Netherlands; ^8^ Department of Urology Netherlands Cancer Institute—Antoni van Leeuwenhoek Hospital Amsterdam The Netherlands; ^9^ Department of Urology, Caritas St. Josef Medical Center University of Regensburg Regensburg Germany

**Keywords:** bladder carcinoma, clonality, upper urinary tract carcinoma, urothelial carcinoma

## Abstract

The risk of developing urothelial carcinoma of the bladder (UCB) in patients treated by radical nephroureterectomy (RNU) for an upper urinary tract urothelial carcinoma (UTUC) is 22% to 47% in the 2 years after surgery. Subject of debate remains whether UTUC and the subsequent UCB are clonally related or represent separate origins. To investigate the clonal relationship between both entities, we performed targeted DNA sequencing of a panel of 41 genes on matched normal and tumor tissue of 15 primary UTUC patients treated by RNU who later developed 19 UCBs. Based on the detected tumor‐specific DNA aberrations, the paired UTUC and UCB(s) of 11 patients (73.3%) showed a clonal relation, whereas in four patients the molecular results did not indicate a clear clonal relationship. Our results support the hypothesis that UCBs following a primary surgically resected UTUC are predominantly clonally derived recurrences and not separate entities.

## Abstract 2

**What's new?**

Patients treated by radical nephroureterectomy for upper urinary tract cancer have an increased risk of developing bladder carcinoma following surgery. It remains unclear, however, whether the upper urinary tract cancer and subsequent bladder carcinoma are clonally related or have separate origins. This targeted DNA sequencing study shows that almost 75% of patients have tumors that are clonally related, suggesting that seeding of tumor cells is the main mechanism of bladder carcinoma development following radical nephroureterectomy. This result underscores the need to minimalize the risk of seeding during surgery and/or diagnostic ureterorenoscopy plus biopsy, and to apply peri‐operative intravesical instillations with chemotherapy.

**Abbreviations**
AI: allelic imbalance
Indels: insertion/deletion
LS: Lynch syndrome
NGS: next‐generation sequencing
SNP: single‐nucleotide polymorphism
SNV: single‐nucleotide variants
TCGA: The Cancer Genome Atlas
UCB: urothelial carcinoma of the bladder
UTUC: upper urinary tract urothelial carcinoma
VAF: variant allelic frequency

## 1 INTRODUCTION

Patients undergoing radical nephroureterectomy (RNU) for upper urinary tract urothelial carcinoma (UTUC) have 22% to 47% risk of developing a subsequent urothelial carcinoma of the bladder (UCB) within 2 years.^1^ Two hypotheses have been proposed for this increased risk. Firstly, the entire urinary tract of patients with urothelial carcinoma undergoes a “field change,” priming the tissue for independent transformations.^2^ Upper and lower tract tumors therefore develop independently from one another and are not clonally related. Secondly, by intraluminal seeding or intraepithelial spread, cancer cells from the primary UTUC implant in the bladder wall and develop into a UCB resulting in clonally related tumors.^3^ Recently, we performed a systematic review of the literature on the clonal relationship between UTUC and paired UCB and found that 94% of the cases originated from the same progenitor cell.^4^ However, the molecular techniques used differed largely over time and research groups, plus only a limited number of studies used comprehensive large‐scale DNA sequencing techniques, which enables more conclusive assessment of a clonal relation between these two entities.

In our study, we used targeted DNA next‐generation sequencing (NGS) to analyze the clonal relationship of primary UTUC and subsequent UCB in patients treated with an RNU based on shared genomic alterations.

## 2 MATERIALS AND METHODS

### 2.1 DNA extraction

Tumor hematoxylin and eosin slides were reviewed by an expert genitourinary pathologist (GvL) and regions containing ≥50% tumor cells were selected for DNA isolation (Supplementary Table 2). Tumor and corresponding normal tissue sections were manually microdissected in 5% Chelex 100 Resin (Bio‐Rad, Hercules, CA) cell lysis solution (Promega, Madison, WI). DNA was extracted by proteinase K (Roche, Mannheim, Germany) digestion at 56°C. Proteinase K was inactivated for 10 minutes at 95°C after which the samples were centrifuged for 5 minutes at 14000 rpm to collect cell debris and chelexresin. Finally, DNA was collected into new tubes and the concentration was measured by using a Qubit 2.0 fluorometer (Thermo Fisher Scientific, Waltham, MA), as described by the manufacturer.

### 2.2 Next‐generation targeted sequencing

For targeted NGS, a custom‐made cancer panel was designed using the AmpliSeq designer (Thermo Fisher Scientific, Waltham, MA). This panel comprised 330 amplicons covering 41 genes, multiple hotspot regions in various cancer‐related genes and 154 single nucleotide polymorphisms in multiple tumor suppressor regions to detect copy number variations (Table 2 and Supplementary Table 3).^5^, ^6^, ^7^ NGS was performed with the Ion Torrent platform using supplier's materials and protocols (Thermo Fisher Scientific). Median coverage depths were 1994x for UTUC, 1712x for UCB and 1914x for the adjacent normal tissue. Libraries were made using the Ion AmpliSeq Library Kit plus–384 LV, template was prepared with the Ion 510/520/530 Chef kit and sequencing was performed on a 530‐chip using the Ion S5 system. Data were analyzed using SeqPilot (JSI medical systems). To correct for potential germline mutations, NGS was also performed on DNA isolated from matched nonmalignant kidney tissue. The final tumor cell percentage was calculated based on the DNA quality and quantity and the results of the NGS.

### 2.3 Genomic alterations

**FIGURE 1:**
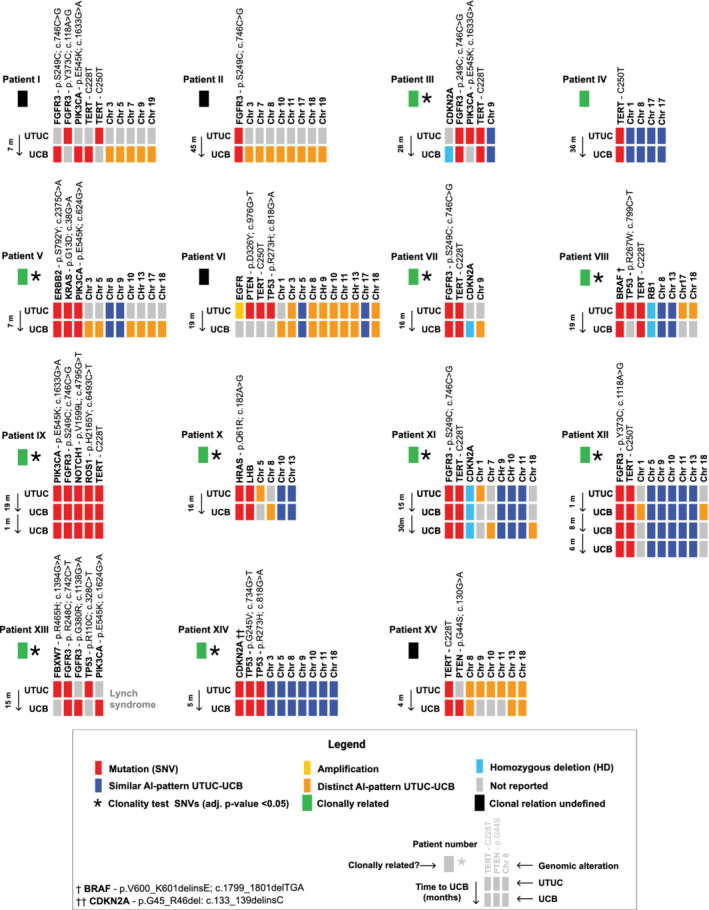
Assessment of the clonal relation of 15 primary UTUC and 19 subsequent UCBs based on (non)shared tumor‐specific genomic alterations between both entities detected by next‐generation sequencing. Additional transcriptomic profiling based on mRNAseq data is included for patients X, XI, XII and XIV (NU, normal ureteric tissue; UCB, urothelial carcinoma of the bladder; UTUC, upper urinary tract urothelial carcinoma) [Color figure can be viewed at wileyonlinelibrary.com]

A visual inspection by an experienced technician (ICM) and clinical scientist (HJD) in molecular pathology making use of Torrent Variant Caller and SNPitty was carried out to identify the genomic alterations.^6^ These genomic alterations were stored in VCF format.^6^, ^8^ Figure 1 summarizes all detected genomic alterations; single‐nucleotide variants (SNVs), indels, allelic imbalance (AI), amplifications and homozygous deletions. For AI analysis, single nucleotide polymorphisms with a total coverage of >100 reads were included. For any informative SNP without AI, a variant allele frequency (VAF) of 0.5 was expected. With a VAF of <0.5 (relative loss of variant allele) or a VAF of >0.5 (relative loss of reference allele), AI was indicated.^9^

### 2.4 Clonality assessment

A possible clonal relationship between UTUC and subsequent UCB(s) was assessed by interrogating all SNVs including synonymous mutations, amplifications, indels and supportive information on AI. To identify if a mutation that was reported in one sample but not in the paired other sample because of insufficient quality reads or absence of that mutation, the following steps were undertaken. A list of all mutations reported in one patient (UTUC and UCBs) was gathered. For every specific position, reads for normal and tumor samples (Phred quality score above ≥15) were subtracted from the BAM files using the bam2R function from the deepSNV (v1.30.0) R package.^10^ Only sites where all samples (tumor and normal) reported a minimum total reads of 30x were included for clonality analysis. The total number of reads was the sum of reference reads plus alternative reads. The VAF from normal tissue samples (VAF_N_) was used as reference to determine SNVs. SNVs and indels were identified when VAF_N_ < 0.10 and VAF_T_ > 0.10. Three samples, the UCBs from Patients II, V and VI, showed some degree of DNA degradation, and the VAF_T_ threshold value was increased to 0.30 to discard most of the false positives with very low VAF_T_.

The probability of a clonal relationship between UTUC and UCB samples from the same patient was evaluated following the clonality test approach developed by Ostrovnaya et al.^11^ The test was performed on all SNVs and indels. As described by Mauguen et al, the clonality test based on SNVs and indels was performed using the mutation reference data set for bladder cancer from the *TCGA* study.^12^ More specifically, frequencies of specific SNVs are assumed to be known. The frequency *f = x/n*, where *x* is the number of tumors with a specific SNV and *n* is the total number of tumors based on *n* = 411 bladder cancer tumors from the *TCGA* cohort. Note that hotspot mutations would have high frequencies and rare mutations would have very low frequencies. When mutations have not been reported in the *TCGA* data set (in case of indels and rare SNVs), the frequency of these mutations was estimated as *f = m*/(*n + m*), where *m* is the number of patients carrying that specific SNV or indel. The frequencies of hotspot mutations in *TERT* promoter (*pTERT*) have not been included in the *TCGA* data set. We completed the data set by adding reported frequencies of *pTERT* C228T (64%) and C250T (13%) mutations from a study by Allory et al.^13^ Based on the marginal frequency of all SNVs and indels, the likelihood ratio test was applied to estimate the probability of a clonal origin of the paired UTUC and UCB.^11^ *P* values were adjusted with the Benjamin and Hochberg method and adjusted *P* values <.05 were considered significant.

## 3 RESULTS

In total, 15 patients with primary UTUC, treated by RNU, who subsequently developed 19 UCBs, treated by transurethral resection of the bladder, were included. Patient, treatment and tumor characteristics of the study population are listed in Table 1 and Supplementary Table 1. Shared genomic variants revealed that UTUC and paired UCB(s) were clonally related in 11 of 15 patients (73.3%) (Figure 1). No significance (*P*_Adj_ = .086) was found for the single‐shared *TERT* (C250T) mutation in Patient IV; however, comparable AI patterns supported clonal origin. Patient XIII, diagnosed with Lynch syndrome (LS), only shared a *Fibroblast Growth Factor Receptor* (*FGFR*)‐3 mutation (p.R248C; c.742C>T) between both tumors. However, as this mutation only occurs in less than 1% of urothelial carcinoma, a clonal relationship remained statistically significant (*P*_Adj_ = .025). Patients II and XV also exhibited only a single‐shared mutation between both tumors, but as these alterations are common hotspot mutations in urothelial carcinoma, the presence in both entities did not unambiguously reflect a clonal relation. In Patients I and VI, we did not observe any shared somatic mutations, so could not support a clonal relationship.

**TABLE 1:** Patient, treatment and tumor characteristics of 15 patients diagnosed with a primary upper urinary tract urothelial carcinoma and a subsequent urothelial carcinoma of the bladder

Variable		Variable	
**Patient characteristics, n = 15**			
Male sex—no. (%)	8 (53.3%)	Smoking status	
Age, years, median (IQR)	67 (12.5)	Never	3 (20.0%)
		Former	9 (60.0%)
		Current	3 (20.0%)
**Treatment characteristics, n = 15**			
Preoperative URS—no. (%)	10 (66.7%)	Hospital that performed RNU—no. (%)	
Bladder cuff removal—no. (%)	10 (66.7%)	Erasmus Medical Center, Rotterdam	7 (46.7%)
Perioperative systemic chemotherapy—no. (%)	0 (0.0%)	Netherlands Cancer Institute, Amsterdam	7 (46.7%)
Perioperative intravesical instillation with chemotherapy—no. (%)	2 (13.3%)	Radboud University Center, Nijmegen	1 (6.6%)
**UTUC characteristics, n = 15**			
Lateralization—no. (%)			
Left	7 (46.7%)		
Right	8 (53.3%)		
Localization—no. (%)			
Renal pelvis	9 (60.0%)		
Ureter	6 (40.0%)		
Pathological T‐stage—no. (%)			
pTa	9 (60.0%)		
pT1	1 (6.7%)		
pT2	3 (20.0%)		
pT3	2 (13.3%)		
Tumor grade (WHO 1973)—no. (%)			
Grade 1	1 (6.7%)		
Grade 2	9 (60.0%)		
Grade 3	5 (33.3%)		
Tumor grade (WHO 2004/2016)—no. (%)			
Low grade	5 (33.3%)		
High grade	10 (66.7%)		
Pathological N‐stage—no. (%)			
pNx	14 (93.3%)		
pN0	1 (6.7%)		
**UCB characteristics, n = 19**		**Time to UCB (months), median (IQR)**	**16.0 (11.5)**
Pathological T‐stage—no. (%)			
pTis	2 (10.5%)		
pTa	15 (79.0%)		
pT1	2 (10.5%)		
Tumor grade (WHO 1973)—no. (%)			
Grade 1	2 (10.5%)		
Grade 2	10 (52.6%)		
Grade 3	7 (36.8%)		
Tumor grade (WHO 2004/2016)—no. (%)			
Low grade	8 (42.1%)		
High grade	11 (57.9%)		

Abbreviations: IQR, interquartile range; RNU, radical nephroureterectomy; TNM stage, based on seventh TNM classification of malignant tumors; UCB, urothelial carcinoma of the bladder; URS, ureterorenoscopy; UTUC, upper urinary tract urothelial carcinoma.

**TABLE 2:** Genes included in the next‐generation DNA targeted sequencing panel

**Gene**	**Exons covered**	**Gene or region**	**Numbers of SNPs included**
*CDKN2A*		Chr1p	11 SNPs
*PTEN*		Chr8p	9 SNPs
*TP53*		Chr7	9 SNPs
*AKT1*	Exon 3	Chr19q	9 SNPs
*ALK*	Exons 20, 22‐25	*APC*	9 SNPs
*Amel_X*	Not applicable	*ARID1A*	8 SNPS
*Amel_Y*	Not applicable	*ATM*	9 SNPs
*APC*	Exon 14	*BRCA1*	9 SNPs
*ARAF*	Exon 7	*BRCA2*	9 SNPs
*BRAF*	Exons 11, 15	*CDKN2A*	9 SNPs
*CHEK2*	Exons 4, 5, 12, 13	*FHIT*	9 SNPs
*CTNNB1*	Exons 3, 7, 8	*PTEN*	9 SNPs
*EGFR*	Exons 18‐21	*RB1*	9 SNPs
*ERBB2*	Exons 19‐21	*SMAD4*	9 SNPs
*EXH2*	Exon 16	*STK11*	9 SNPs
*FBXW7*	Exons 9, 10	*TP53*	9 SNPs
*FGFR1*	Exons 7, 9	*VHL*	9 SNPs
*FGFR2*	Exons 7, 9		
*FGFR3*	Exons 7, 9		
*FOXL2*	Exon 3		
*GNA11*	Exons 4,5		**Total number of amplicons**
*GNAS*	Exons 8, 9		330
*HRAS*	Exons 2–4		
*IDH1*	Exon 4		
*IDH2*	Exon 4		
*KIT*	Exons 8, 9, 11, 13, 14, 17		
*KRAS*	Exons 2–4		
*MAP2K1*	Exons 2, 3		
*MET*	Exons 2, 14, 19		
*MYD88*	Exon 5		
*NOTCH1*	Exons 26, 27		
*NRAS*	Exons 2–4		
*PDGFRa*	Exons 12, 14, 18		
*PIK3CA*	Exons 10, 21		
*POLD1*	Exons 12		
*POLE*	Exons 9, 13		
*RAF1*	Exon 7		
*RET*	Exons 11, 16		
*RNF43*	Exons 3, 4, 9		
*SMAD4*	Exons 3, 9, 12		
*STK11*	Exons 4, 5, 8		
*TERT promoter*	Promoter region		

*Note:* Diagnostic V5.1 next‐generation sequencing panel. Erasmus Medical Center, Rotterdam.

## 4 DISCUSSION

Studies that used large‐scale sequencing techniques to assess the clonality of UTUC and paired UCB are scarce. In 2017, Du et al analyzed five patients with synchronous UTUCs (n = 9) and UCBs (n = 4) by whole exome sequencing.^14^ Tumors were clonally related in only two patients; a lower proportion than we found in the present study. Exposure to aristocholic acid was linked to tumor development in all five patients, which possibly affected the entire urothelium leading to field cancerization. Audenet et al reported on a cohort of 29 patients with paired UTUC and UCB, and found all tumors to be clonally related, although this cohort also included patients with a history of primary UCB and some exhibited synchronous tumors.^15^ In the present study, we only included patients with primary UTUC and metachronous UCB(s); an approach which more accurately reflects the natural course of surgically treated UTUC patients.

The observed differences in cohort clonality may reflect patient idiosyncrasies, but also highlight remaining technical challenges. Targeted panels do not to cover all genomic aberrations, so clonality might have been underestimated in our study. Shared alterations could have been missed due to the extent of this panel, which increases the likelihood that the UCBs, which were found not to be clonally related, could have been clonally derived recurrences. Reductions in sequencing cost, and the application of whole genome or exome RNA‐DNA sequencing, offer opportunities to expand the search for clonal markers. Tumor heterogeneity may be an alternative explanation for the ~25% of paired tumors we analyzed which did not appear clonally related: it cannot be unambiguously excluded that clonality was masked for these tumors. Furthermore, as a relatively rare cancer, there are limited data on UTUC‐specific mutation frequencies. Pertinently, recent work proposed enrichment of the *FGFR3* p.R248C amino acid substitution in LS‐linked UTUC, and so it is debatable whether this shared alteration alone indicates a clonal relationship in Patient XIII. Particularly when LS patients may exhibit a higher probability of developing multiple urinary tract tumors.^16^ Notwithstanding these limitations, our observation that almost 75% of the paired tumors were clonally related strongly suggests that seeding of tumor cells from the upper urinary tract to the bladder represents the most important mechanism of UCB development following RNU. Importantly, three patients in our cohort developed multiple subsequent UCBs, and all tumors were clonally related to the primary UTUC, which further supports the mechanism of seeding of tumor cells.

## 5 CONCLUSIONS

The results of our study underscore the rationale to (a) minimalize the risk of seeding of tumor‐cells during RNU; (b) carefully consider the need for diagnostic work‐up by ureterorenoscopy and biopsy, which can dissociate cancer cells, and (c) apply perioperative intravesical instillations with chemotherapy to kill cancer cells floating in urine. Large‐scale genomic characterization of a properly selected cohort of UTUC and paired UCB using unbiased sequencing techniques will overcome the aforementioned limitations and will further clarify clonal relationships between in‐patient upper and lower tract urothelial carcinomas.

## CONFLICT OF INTEREST

J. L. Boormans reports on consultancy work for MSD, Janssen, Ambu, Health care and Ismar, during the conduct of the study; and received a research grant from Decipher Biosciences. All other authors report no conflict of interest.

## ETHICS STATEMENT

No approval was required for our study.

## Supporting information


**Supplementary Table 1** Tumor characteristics.
**Supplementary Table 2**: Tumor cell percentage.
**Supplementary Table 3**: Reference ID's SNPs.

## Data Availability

The data that support the findings of this study are available from the corresponding author upon reasonable request.
