# Supplementary material for: The clonal relation of primary upper urinary tract urothelial carcinoma and paired urothelial carcinoma of the bladder
Source: Int J Cancer. 2020 Oct 13;148(4):981–7. doi: 10.1002/ijc.33327 (PMC7821318; doi:10.1002/ijc.33327)
Supplement: Supplementary file 1 — Supplementary Table 1 Tumor characteristics. Supplementary Table 2: Tumor cell percentage. Supplementary Table 3: Reference ID's SNPs. [file IJC-148-981-s001.pdf]

The clonal relation of primary upper urinary tract urothelial carcinoma and paired urothelial carcinoma of the bladder

Thomas van Doeveren[a,1], Jose A. Nakauma-Gonzalez[a,1,2,3], Andrew S. Mason[4,5], Geert J.L.H. van Leenders[6], Tahlita C.M. Zuiverloon[1], Ellen C. Zwarthoff[6], Isabelle C. Meijssen[6], Angelique C. van der Made[6], Antoine G. van der Heijden[7], Kees Hendricksen[8], Bas W.G. van Rhijn[8,9], Charlotte S. Voskuilen[8], Job van Riet[1,3], Winand N.M. Dinjens[6], Hendrikus J. Dubbink[6], Harmen J.G. van de Werken[1,3], Joost L. Boormans[1]

Content

- **Supplementary Table 1**: Tumor characteristics.
- **Supplementary Table 2**: Tumor cell percentage.
- **Supplementary Table 3**: Reference ID's SNPs.

Supplementary Table 1. Pathological T-stage, WHO grade 1973 and 2004/2016, and time to recurrence from RNU in months of 15 upper tract urothelial carcinomas and 19 paired urothelial carcinomas of the bladder

	UTUC	UCB I (months*)	UCB II (months)	UCB III (months)
Patient I	TaG1-low grade	TaG2-low grade (7)		
Patient II	TaG2-low grade	TaG1-low grade (45)		
Patient III	TaG2-low grade	TaG2-low grade (28)		
Patient IV	TaG2-high grade	TaG3-high grade (36)		
Patient V	TaG2-low grade	TaG2-low grade (7)		
Patient VI	T2G3-high grade	TisG3-high grade (19)		
Patient VII	T3G2-low grade	T1G2-low grade (16)		
Patient VIII	TaG2-high grade	TaG2-high grade (19)		
Patient IX	TaG2-high grade	TaG2-high grade (19)	TaG2-high grade (20)	
Patient X	T3G3-high grade	TaG3-high grade (16)		
Patient XI	TaG2-high grade	TaG1–low grade (15)	TaG3-high grade (45)	
Patient XII	T1G3-high grade	TaG3-high grade (1)	TaG2-low grade (9)	TaG2-low grade (15)
Patient XIII	TaG3-high grade	TaG2-high grade (15)		
Patient XIV	T2G2-high grade	T1G3-high grade (6)		
Patient XV	T2G3-high grade	TisG3-high grade (4)		

*months to recurrence from RNU; UTUC = upper urinary tract urothelial carcinoma; UCB = urothelial carcinoma of the bladder

Supplementary Table 2. The actual tumor cell percentage based on the *Next Generation DNA targeted sequencing* data of 15 upper tract urothelial carcinomas and 19 paired urothelial carcinomas of the bladder

Tumor cell percentage				
	**UTUC**	**UCB I**	**UCB II**	**UCB III**
Patient I	70%	60%		
Patient II	70%	70%		
Patient III	70%	70%		
Patient IV	70%	70%		
Patient V	80%	50%		
Patient VI	60%	50%		
Patient VII	90%	90%		
Patient VIII	90%	90%		
Patient IX	80%	80%	80%	
Patient X	70%	80%		
Patient XI	80%	80%	80%	
Patient XII	80%	70%	70%	70%
Patient XIII	80%	70%		
Patient XIV	90%	80%		
Patient XV	80%	50%		

UTUC = upper urinary tract urothelial carcinoma; UCB = urothelial carcinoma of the bladder

Supplementary Table 3 Reference ID's of the 154 SNPs and corresponding chromosome number used in the Next Generation Sequencing panel

Reference SNP ID numbers							
Chrom.	Reference ID	Chrom.	Reference ID	Chrom.	Reference ID	Chrom.	Reference ID
1	Rs72901775	5	Rs163454	10	Rs2248456	17	Rs11658073
1	Rs7546616	5	Rs2019720	10	Rs1426618	17	Rs1905338
1	Rs12567277	5	Rs2431512	10	Rs2274312	17	Rs3760386
1	Rs9439532	5	Rs448475	10	Rs10887863	17	Rs9915489
1	Rs9434673	5	Rs13190040	11	Rs11212118	17	Rs8070179
1	Rs622662	5	Rs7727449	11	Rs10431058	17	Rs799905
1	Rs6673156	5	Rs6871811	11	Rs11212459	17	Rs11655135
1	Rs7663	7	Rs190	11	Rs228589	17	Rs231478
1	Rs1127818	7	Rs2028209	11	Rs645485	17	Rs7217858
1	Rs10794522	7	Rs983613	11	Rs227093	18	Rs7241428
1	Rs2445635	7	Rs2072453	11	Rs949286	18	Rs1893489
1	Rs383913	7	Rs730437	11	Rs2162156	18	Rs2427777
1	Rs11247593	7	Rs6950826	11	Rs625040	18	Rs620898
1	Rs11247594	7	Rs2037588	13	Rs9602144	18	Rs17736674
1	Rs7504	7	Rs2057932	13	Rs9548593	18	Rs7244552
1	Rs3813795	7	Rs6957957	13	Rs7334588	18	Rs2445441
1	Rs2504786	8	Rs1564480	13	Rs2126043	18	Rs9951319
1	Rs6564	8	Rs9644053	13	Rs9534262	18	Rs12606702
1	Rs157208	8	Rs900779	13	Rs693963	19	Rs2312104
3	Rs2600160	8	Rs68063696	13	Rs7993153	19	Rs55913760
3	Rs2542381	8	Rs2249102	13	Rs450789	19	Rs1683561
3	Rs7613920	8	Rs7013223	13	Rs5011113	19	Rs7253869
3	Rs263411	8	Rs10112431	13	Rs1572871	19	Rs4995472
3	Rs696219	8	Rs13276054	13	Rs1326466	19	Rs4807072
3	Rs33269	8	Rs6557686	13	Rs7338119	19	Rs12460075
3	Rs892607	9	Rs10964823	13	Rs7994141	19	Rs1061828
3	Rs2697161	9	Rs614647	13	Rs9568036	19	Rs2074552
3	Rs1062246	9	Rs10757225	13	Rs9535032	19	Rs7283
3	Rs12491197	9	Rs10757261	13	Rs9595961	19	Rs33841
3	Rs13317082	9	Rs3814960	13	Rs7987258	19	Rs1291
3	Rs28597374	9	Rs10965215	13	Rs2407610	19	Rs166539
3	Rs67777617	9	Rs828582	17	Rs4796561	19	Rs10113
3	Rs7646323	9	Rs10511705	17	Rs90951	19	Rs6521
3	Rs11130822	9	Rs7852081	17	Rs4796409	19	Rs193040
3	Rs9816766	10	Rs10749542	17	Rs1050541	19	Rs10217
3	Rs11708109	10	Rs3802665	17	Rs7141	19	Rs10448
3	Rs7647357	10	Rs187893001	17	Rs62062589		
5	Rs9784704	10	Rs4933453	17	Rs8073033		
5	Rs30341	10	Rs7076964	17	Rs6503098		

Chrom. = Chromosome
